# A120 ENDOSCOPIC CLOSURE TECHNIQUES FOLLOWING ACUTE IATROGENIC PERFORATION -- AN UPDATED SYSTEMATIC REVIEW

**DOI:** 10.1093/jcag/gwac036.120

**Published:** 2023-03-07

**Authors:** J Buttar, R Enns, E Lam, N Shahidi

**Affiliations:** 1 Internal Medicine; 2 Adult Gastroenterology; 3 Adult Gastroenterology - Clinical Associate Professor, University of British Columbia, Vancouver, Canada

## Abstract

**Background:**

Iatrogenic perforation is arguably the most feared adverse event associated with endoscopy. Current American Society for Gastrointestinal Endoscopy (ASGE) and European Society of Gastroenterology Endoscopy (ESGE) guidelines recommend endoscopic closure as the first-line treatment strategy. Historically, this has been achieved using through-the-scope clips (TTSC). Given the emergence of alternative endoscopic closure techniques including over-the-scope clips (OTSC) and endoscopic suturing, we sought to provide an updated review of the literature.

**Purpose:**

To review endoscopic closure techniques following iatrogenic perforation during screening or therapeutic endoscopy.

**Method:**

Based on the Preferred Reporting Items for Systematic Reviews and Meta-analyses (PRISMA) Guidelines, an electronic search of MEDLINE and EMBASE from June 1^st^, 1946 – Oct 10^th^, 2022 was performed. Inclusion criteria was limited to English full-text original citations, with case reports, and cohorts with < 3 patients excluded. Our primary objective was to assess complete defect closure after attempted endoscopic treatment. Outcomes were stratified by modality (TTSC, OTSC, endoscopic suturing) and chronologically based on a previous well received systematic review.

**Result(s):**

A total of 2549 citations were identified in our electronic search, of which 34 were included representing 830 perforations. Overall, successful endoscopic closure was achieved in 763 cases (91.9%). When stratified by endoscopic closure techniques, range estimates for successful endoscopic closure was 71% – 100%, 57% - 100%, and 100% for TTSC, OTSC and endoscopic suturing respectively. When stratifying chronologically, an improvement in TTSC closure was identified.

**Conclusion(s):**

Endoscopic defect closure, including TTSC, OTSC and endoscopic suturing, are effective in the management of iatrogenic perforations with increasing TTSC performance over time. It remains the primary treatment strategy for iatrogenic perforation.

**Please acknowledge all funding agencies by checking the applicable boxes below:**

None

**Disclosure of Interest:**

None Declared

